# Negative-pressure wound therapy is effective for peritoneal dialysis catheter exit-site management in the early postoperative period

**DOI:** 10.1038/s41598-021-03878-5

**Published:** 2022-01-07

**Authors:** Haruna Fukuzaki, Junichiro Nakata, Shuko Nojiri, Yuki Shimizu, Toshiki Kano, Yuka Shirotani, Takuya Maeda, Nao Nohara, Hiroaki Io, Yusuke Suzuki

**Affiliations:** 1 Department of Nephrology, Juntendo University Faculty of Medicine, Tokyo, Japan; 2 Medical Technology Innovation Center, Juntendo University, Tokyo, Japan; 3 Department of Nephrology, Juntendo University Nerima Hospital, Tokyo, Japan

**Keywords:** Medical research, Nephrology

## Abstract

Peritoneal dialysis (PD) catheter exit-site care is critically important for the prevention of catheter-related infections (CRIs) and subsequent peritonitis. The postoperative management of the site is particularly essential because it has an open wound that is always adjacent to a PD catheter tube. This study aimed to examine the effectiveness of negative-pressure wound therapy (NPWT) for postoperative PD catheter exit sites. Thirty patients with end-stage renal disease who underwent simultaneous PD catheter insertion and exit-site formation were randomly assigned to receive NPWT (NPWT group) or conventional dressing (non-NPWT group) for the first seven postoperative days. The exit-site scores on the seventh postoperative day was lower in the NPWT group than in the non-NPWT group (*p* = 0.0049). Analysis of variance F statistic for the effect of NPWT over 180 days was highly significant (11.482595, *p* = 0.007). There were no statistically significant differences between the time to first CRI and PD-related peritonitis between the two groups. There was one case of CRI with relapsing peritonitis and catheter loss in the non-NPWT group. These findings demonstrate the association between NPWT and low exit-site score. NPWT can be recommended for the management of PD catheter exit sites in the early postoperative period.

## Introduction

Peritoneal dialysis (PD)-related peritonitis is one of the most common and most serious complications of PD^1^. It is reported to be a direct or major contributing cause of death in about 16% of PD patients^2,3^. Moreover, peritonitis is a major cause of discontinuation of PD and change to long-term hemodialysis^2^. Exit-site infections (ESIs) and catheter-tunnel infections, which are usually described as catheter-related infections (CRIs), are major predisposing factors of PD-related peritonitis^4,5^. National and international consensus guidelines, which are issued by the International Society for Peritoneal Dialysis (ISPD), focus on identifying the best strategies for the prevention and treatment of CRIs and PD-related peritonitis^1,5,6^. Many of the guidelines, which provide strong recommendations, are controversial or supported by limited evidence. However, it is clear from the guidelines that PD catheter exit-site care is critically important for the prevention of CRIs and PD-related peritonitis.

A PD catheter exit site is an open wound made during operation using a tunneler, and it is always in contact with a catheter tube. Retardation of exit-site healing in the postoperative period sometimes leads to early CRIs. Negative-pressure wound therapy (NPWT), first developed in the early 1990s^7,8^, is a noninvasive wound healing technique used for a wide range of complex wounds^9^. NPWT involves the delivery of partial vacuum with suction through a special dressing and therapy unit, which creates an environment that promotes wound healing. In current clinical settings, NPWT is used for a variety of wound types, including open traumatic wounds^10^, skin grafts^11^, burns^12^, venous leg ulcers^13^, diabetic foot ulcers^14^, and surgical site infections^15^.

A study by Mori et al*.* suggested that NPWT is effective for PD catheter exit sites^16^; however, this was an observational study. The present study aimed to evaluate the effectiveness of NPWT for postoperative PD catheter exit-site care via a randomized open-label trial.

## Results

### Patient characteristics

**Figure 1:**
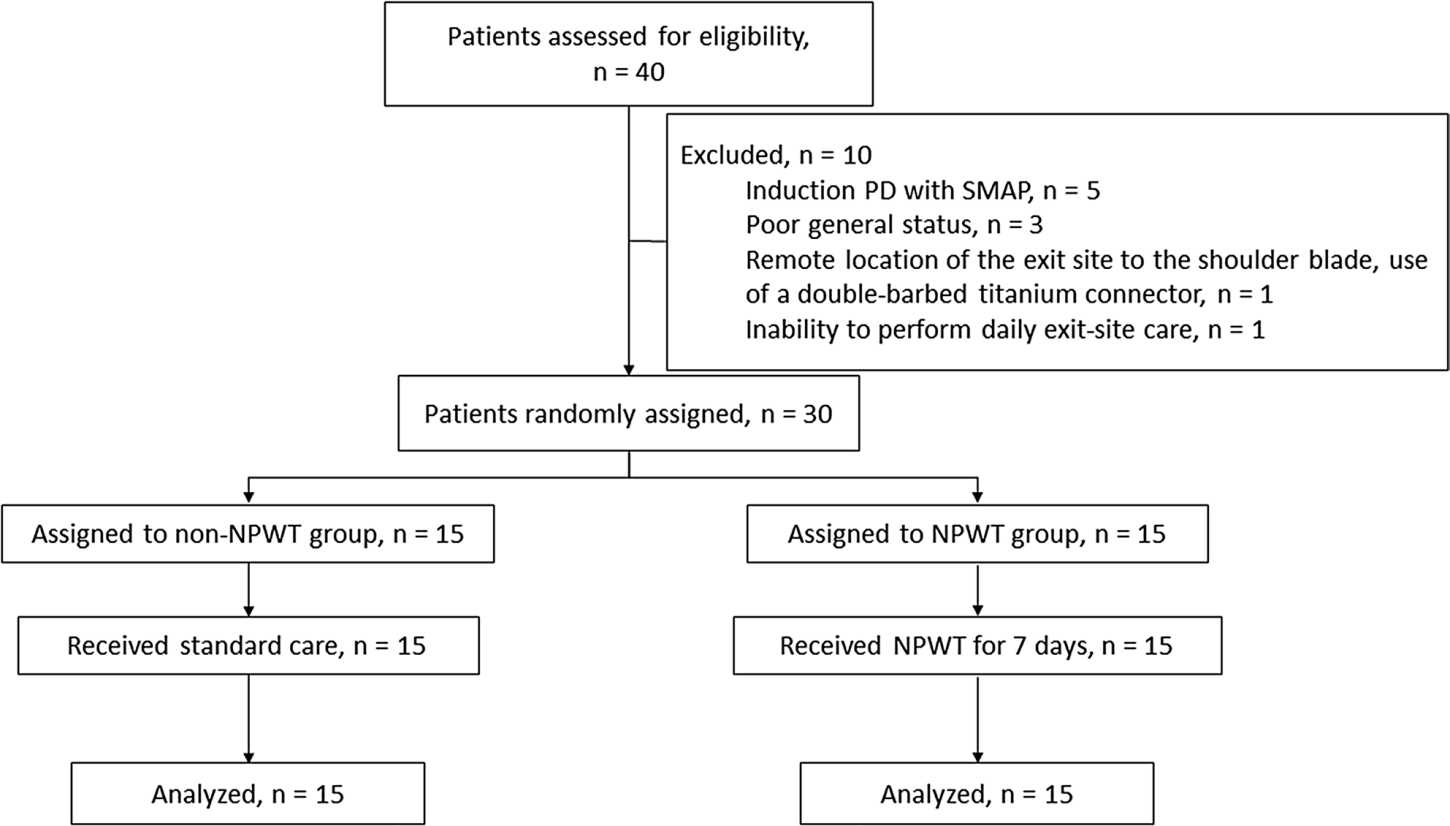
Allocation and course of study participants. PD, peritoneal dialysis; SMAP, stepwise initiation of peritoneal dialysis using the Moncrief–Popovich technique; NPWT, negative-pressure wound therapy.

For this study, 40 patients were assessed for eligibility (Fig. 1). Ten patients were excluded: five patients induced PD with the stepwise initiation of PD using the Moncrief–Popovich technique, three patients had poor general status (end-stage gastric cancer, severe congestive heart failure, and severe infection with sepsis), one patient was considered ineligible (a remote location of the exit site to the shoulder blade using a double-barbed titanium connector), and one patient was unable to perform daily exit-site care. A total of 30 patients (15 patients in each group) were enrolled and analyzed. Patient characteristics are summarized in Table 1. There were no significant differences in age, sex, primary cause of end-stage renal disease, body mass index, smoking habit, and thickness of subcutaneous fat on the abdominal wall between the two groups. Except for postoperative white blood cell count, there were also no significant differences in laboratory data between the two groups.

**Table 1:** Baseline characteristics of the patients.

	Total (n = 30)	Non-NPWT group (n = 15)	NPWT group (n = 15)	*p* value
Age (years)	55.5 (37–94)	54 (37–84)	58 (40–94)	0.9338
Male sex (%, [n])	90.0 (27)	86.7 (13)	93.3 (14)	0.3829
Primary cause of ESRD				
CGN (%, [n])	26.7 (8)	20.0 (3)	33.3 (5)	0.2334
Diabetes (%, [n])	23.3 (7)	20.0 (3)	26.7 (4)	0.3051
Nephrosclerosis (%, [n])	20.0 (6)	20.0 (3)	20.0 (3)	0.3487
Others (%, [n])	30.0 (9)	40.0 (6)	20.0 (3)	0.1592
BMI (kg/m^2^)	22.0 (17.8–34.3)	21.9 (17.8–34.3)	22.1 (18.4–26.9)	0.8357
Currently smoking (%, [n])	16.7 (5)	20.0 (3)	13.3 (2)	0.3352
Thickness of subcutaneous fat on abdominal wall (mm)	17.1 (8.7–43.1)	23.3 (8.8–43.1)	16.7 (8.7–26.1)	0.0970
**Patient data on operation day**				
WBC count (/μL)	4950 (3500–11,400)	5300 (4300–11,400)	4800 (3500–7000)	0.1098
Hb level (g/dL)	10.45 (7.7–13.2)	10.8 (7.7–12.4)	10.1 (7.8–13.2)	0.7871
Alb level (g/dL)	3.6 (2.4–4.6)	3.6 (3.0–4.6)	3.6 (2.4–4.4)	0.9001
eGFR (mL/min/1.73 m^2^)	6.6 (2.8–9.9)	7.4 (2.8–9.9)	5.4 (3.6–9.8)	0.2209
HbA1c level (%)	5.45 (4.6–8.0)	5.5 (5.0–8.0)	5.4 (4.6–7.8)	0.6319
hsCRP level (mg/dL)	0.0735 (0.02–2.445)	0.103 (0.02–2.445)	0.056 (0.02–0.743)	0.2967
BNP level (pg/mL)	85.1 (13.7–1783.9)	59.5 (13.7–1783.9)	95.7 (31.7–475.4)	0.8519
**Patient data on seventh postoperative day**				
WBC count (/μL)	5600 (3300–12,000)	6000 (3700–12,000)	4800 (3300–9600)	0.0309*
hsCRP level (mg/dL)	0.4225 (0.02–10.711)	0.422 (0.02–10.711)	0.452 (0.048–9.758)	0.5897

Data are presented as median (minimum value–maximum value) unless otherwise stated.

NPWT, negative-pressure wound therapy; ESRD, end-stage renal disease; CGN, chronic glomerular nephropathy; BMI, body mass index; WBC, white blood cell; Hb, hemoglobin; Alb, albumin; eGFR, estimated glomerular filtration rate; HbA1c, glycated hemoglobin; hsCRP, high-sensitivity C-reactive protein; BNP, brain natriuretic peptide.

*p < 0.05.

### Procedure outcomes

**Figure 2:**
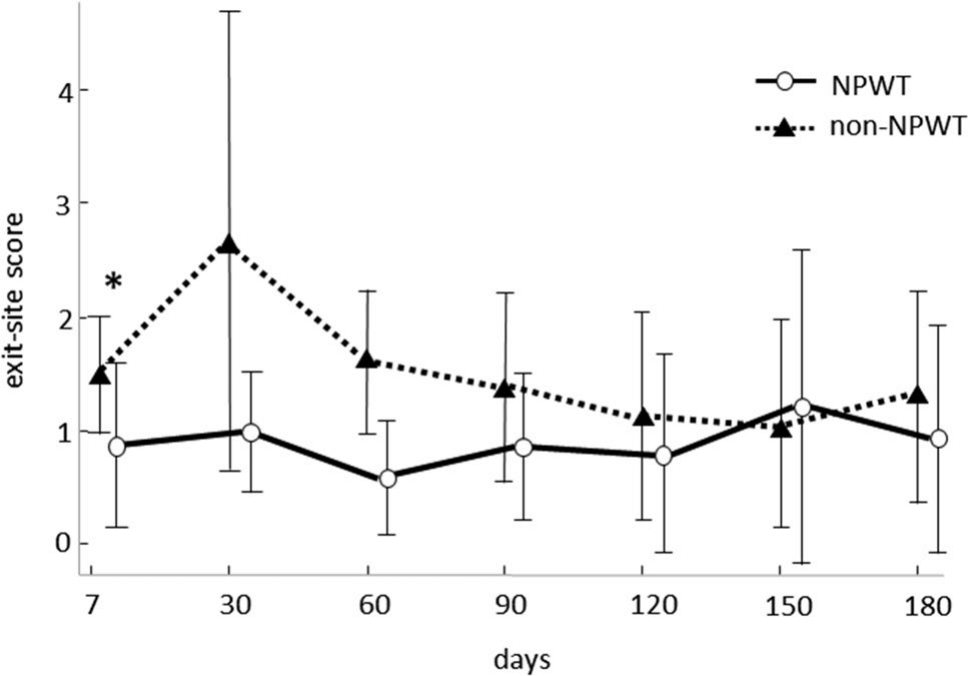
Exit-site scores over 180 days in the NPWT and non-NPWT groups. The exit-site scores on the seventh postoperative day and over 180 days are significantly lower in the NPWT group than in the non-NPWT group (*p* = 0.0049, Mann–Whitney *U* test; *p* = 0.007, analysis of variance, respectively). *p < 0.05. NPWT, negative-pressure wound therapy.

**Figure 3:**
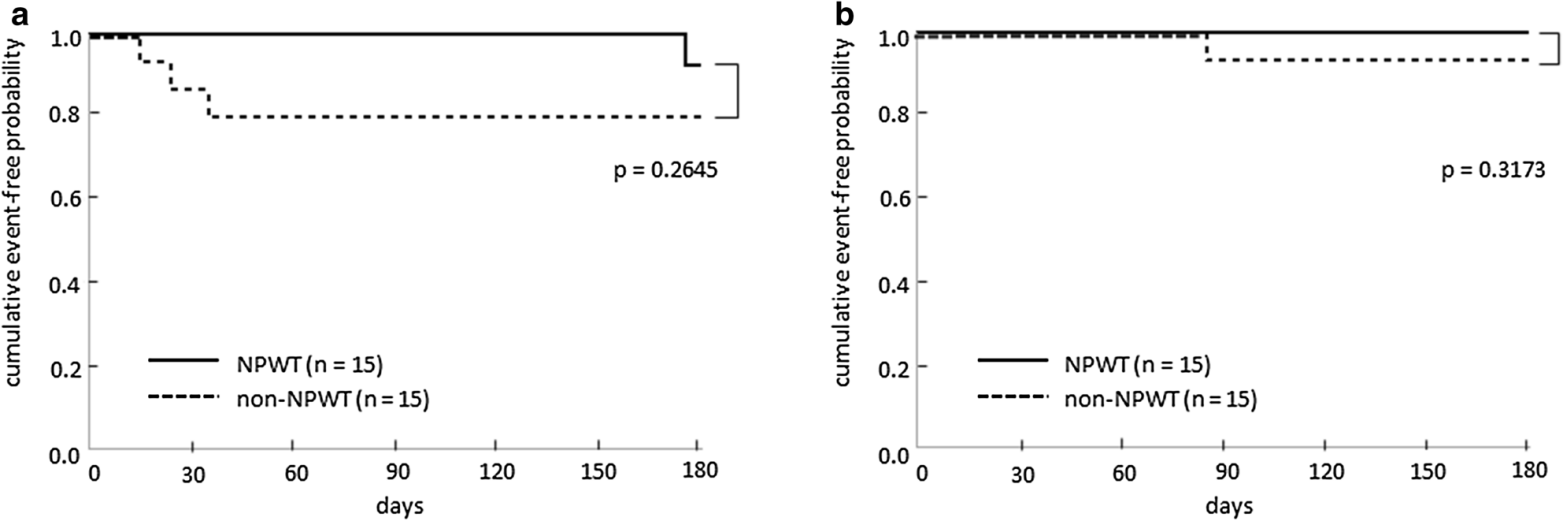
Kaplan–Meier curves of time to (**a**) first catheter-related infection and (**b**) PD-related peritonitis for the NPWT and non-NPWT groups. No statistically significant differences between the two groups are observed for both endpoints (*p* = 0.2645 and *p* = 0.3173, respectively; log-rank test). *p < 0.05. PD, peritoneal dialysis; NPWT, negative-pressure wound therapy.

The procedure outcomes are summarized in Figs. 2 and 3. The exit-site score on the seventh postoperative day was significantly lower in the NPWT group than in the non-NPWT group (1.0 [0–2] and 2.0 [1, 2], respectively, *p* = 0.0049, Mann–Whitney *U* test). Regarding the secondary outcomes, analysis of variance revealed that exit-site score over 180 days after the operation was also statistically lower in the NPWT group than in the non-NPWT group (*p* = 0.007). There were no statistically significant differences in time to first CRI and PD-related peritonitis between the two groups (*p* = 0.2645 and *p* = 0.3173, respectively, log-rank test).

In the non-NPWT group, there was a case of catheter loss due to PD-related infections. The patient presented with ESI a month after operation and had relapsing PD-related peritonitis, which warranted the removal and reinsertion of the PD catheter.

## Discussion

A PD catheter exit site is an open wound made using a tunneler, and it is always adjacent to a catheter tube. Catheters are typically made of smooth silicone rubber, and prolonged exit-site healing sometimes leads to early CRIs. Hence, strategies that prevent delay of exit-site healing and promote exit-site stabilization are essential for PD patients.

Wound bed preparation is a systematic strategy aimed at improving wound management by identifying and removing barriers to wound healing. The prototype of the concept emerged in the 1990s, gradually gained recognition, and developed into a systematized paradigm for the strategy of chronic wound management^17,18^. The concept provides clinicians with a comprehensive approach to removing barriers to healing and stimulating the healing process for maximum benefit. Schultz et al*.* proposed the TIME acronym, which highlights four elements that need to be resolved with regard to wound bed preparation^19^. “T” stands for tissue, “I” stands for inflammation and infection, “M” refers to moisture imbalance, and “E” refers to epithelial edge advancement. According to this concept, adequate management of the postoperative exit site of a PD catheter is necessary to avoid infection, moisture imbalance, and advancement of the wound edges.

In our study, the NPWT group had lower exit-site scores on the seventh postoperative day than the non-NPWT group. The proposed effects of NPWT on postoperative PD catheter exit sites are discussed below. First, the sites are kept hygienic because they are covered with special foam dressings and sealed with clear drape on the operative field. Second, continuous negative pressure removes exudate, resulting in a properly moisturized environment. The PD catheter is blindly directed through a subcutaneous tunnel to the marked site using a tunneler. This procedure frequently causes injury to small vessels in the subcutaneous tissue. In such cases of injury, prolonged bleeding from the exit site often occurs. The system of vacuum with suction is also effective for eliminating infectious wastes at the exit site. Third, the negative pressure causes contraction of the wound edge. Twadroski et al*.* hypothesized that loose fit of skin around the PD catheter would increase the risk of trauma and contamination^20^. NPWT can reduce the surface area of the open wound and lead to early tight fixation. We posit that these three principal mechanisms discussed above great influence the PD catheter exit site as evidenced by the significantly low exit-site scores and the early stabilization of the exit site in the NPWT group. In contrast, the non-NPWT group tended to have high exit-site scores on the seventh postoperative day as evidenced by redness, crusts, and serous drainage. This finding suggests that the exit site is susceptible to trauma due to loose fixation and moisture imbalance caused by the extra exudate, especially after the operation. These factors also paradoxically reveal the usefulness of NPWT for postoperative exit sites.

Regarding secondary outcomes, the NPWT group also had lower exit-site scores over 180 days than the non-NPWT group. However, no statistically significant differences in time to first CRI and PD-related peritonitis were noted between the two groups. These results suggest that, over the long term, exit-site score and the incidence of CRI are independent variables. We hypothesized that daily self-care practice on the exit site has a major impact on long-term CRI and PD catheter-related peritonitis. Although our center ensured that patients underwent a three-week PD training conducted by nursing staff with the appropriate qualifications and experience during their hospitalization, daily PD catheter exit-site care tends to become more personalized as the PD duration increases. Ding et al. reported that the level of adherence to different aspects of exit-site care varies among PD patients^21^. The study found that patient adherence to proper hand hygiene, mask wearing, exit-site observation, secretion examination, and effective communication with PD staff influenced CRI history and exit-site issues. Russo et al. reported that approximately one-third of PD patients needed reinforcements in knowledge and ability to correctly perform PD-related infection control and concluded the retraining needs of patients on PD^22^. As described, educating PD patients to recognize the importance of exit-site assessment, daily self-care, and retraining of the relevant skills must be considered useful for preventing CRIs.

“Down-growth” may also contribute to the long-term acute outbreak of infections. As earlier noted, a PD catheter exit site is an open wound that is always adjacent to a catheter tube. Resistance to infection depends on the formation of a biological barrier formed from tissue growing along a subcutaneous cuff made of polyethylene terephthalate. The epithelium contiguous to the silicone catheter tends to invert toward the cuff, creating a sinus between the tube and the skin. The exit site on which the “down-growth” forms under experimental conditions tends not to have swelling, crust, redness, or pain unless it is infected. This is because a biological barrier composed of epithelium typically covers the catheter tube completely. However, the “down-growth” forms a dead space that promotes accumulation of metabolic products and bacterial colonization, which result in CRIs^23,24^. Although PD catheter exit site “down-growth” has been reported in a few studies, only the study by Yoshino et al. reported that redundant “down-growth” and increase in dead space are associated with insufficient catheter fixation and altered metabolism^25^. It is easy to anticipate that trauma and contamination in the sinus directly increase the risks of CRIs and subsequent peritonitis.

The exit-site scoring system provided by ISPD was originally proposed by Schaefer et al. and applied to pediatric patients^26^. Using this scoring, ESI is diagnosed via ≥ 4 points or through the presence of purulent discharge with or without skin erythema at the catheter–epidermal interface. There has been uncertainty regarding the usefulness of this scoring system, as observed in some recent studies^27,28^. Furthermore, regularly monitoring exit-site score itself cannot predict or prevent CRIs. In contrast, Sangeetha et al.^29^ previously reported that patients with an ISPD exit-site score of 3 points fell in the category of “equivocal” under the Twardowski and Prowant’s exit-site classification and developed peritonitis in 27.2% with catheter loss in all of them, implying that clinicians should be more vigilant about the possibility of developing infection as the score is higher even though it is within 3 points. This reiterates that the scoring system is a valuable scale, and in our trial, it is considered the most universal and objective measurement for verifying the effectiveness of NPWT for postoperative PD catheter exit site. Moreover, maintaining a low exit-site score, i.e., being categorized as “perfect” or “good” under the Twardowski and Prowant’s exit-site classification reflects a CRI-free condition, which may prevent PD-related peritonitis.

The cost of NPWT is USD 90 per day. The high cost may hinder its wide application to a PD catheter exit site. Therefore, it is arguable whether NPWT is a cost-effective therapy for the management of postoperative PD catheter exit site.

To the best of our knowledge, this study is the first randomized open-label trial to examine the effectiveness of NPWT for postoperative PD catheter exit sites. Over the years, many strategies aimed at preventing CRI and subsequent peritonitis have been attempted. Although ISPD guidelines provide some strong recommendations, many of them are controversial or supported by limited evidence. In the study by Mori et al., it was reported that NPWT is effective for early tight fixation to the skin around the catheter^16^; however, the study was observational. Our randomized prospective study shows that NPWT is associated with low exit-site scores in the early postoperative period and early stabilization of the exit site.

Our study has some limitations that should be noted. The required sample size of this study was calculated based on the estimation of statistically significant difference in exit-site scores. Although our study did not reveal statistically significant differences in time to first CRI and PD-related peritonitis between the NPWT and non-NPWT groups, larger samples and longer follow-up durations are necessary for more accurate assessments of PD patients. This is because the incidences of CRI and PD-related peritonitis have decreased considerably; for example, the incidence rate of PD-related peritonitis was reported to be 0.26 episodes/patient-year^30^.

In conclusion, this study is the first randomized open-label trial to assess the effectiveness of NPWT for postoperative exit sites by comparing NPWT with conventional dressing in terms of exit-site score. The NPWT group had significantly lower exit-site scores on the seventh postoperative day and over 180 days than the non-NPWT group. Therefore, we recommend NPWT as an option for early stabilization of the exit site.

## Methods

### Study design

This study was a single-center, randomized, open-label study conducted from November 2017 to April 2021 at Juntendo University Hospital, Tokyo, Japan. The study protocol was approved by the Ethics Committee of Juntendo University Hospital, Tokyo, Japan (approval number: 17–141). In the present study, all procedures involving human participants were performed in accordance with the ethical standards of the institutional and/or national research committee and the 1964 Helsinki declaration and its later amendments or comparable ethical standards. All participants provided written informed consent before enrollment. The study was registered with University Hospital Medical Information Network Clinical Trials Registry on 19/11/2017 (UMIN000030033).

### Participants

Patients scheduled to undergo PD catheter insertion surgery were enrolled in the study. The inclusion criteria were as follows: (1) age ≥ 20 years, (2) planned simultaneous PD catheter insertion and exit-site formation using a tunneler from November 2017 to November 2020, (3) exit-site creation on the abdominal wall, and (4) assumed ability to perform daily exit-site care. The exclusion criteria were as follows: (1) poor general status, (2) refusal to participate in the study, (3) perceived ineligibility, and (4) PD catheter implantation with stepwise initiation of PD using the Moncrief–Popovich technique, which entails the implantation of a catheter, with its end buried in subcutaneous tissue, until the initiation of PD^31^.

Eligible patients for the trial were randomized via a simple computer-generated randomization chart into those who would undergo NPWT (NPWT group) or conventional dressings (non-NPWT group) of the exit site (Fig. 1).

### Surgical procedures

The proposed exit site on the abdominal wall was examined preoperatively with the patient lying down, standing up, and sitting up. The exit site was also checked to ensure that it is sufficiently visible for easy daily exit-site care and that there is no interference with skin wrinkles or the belt line.

The PD catheter insertion surgery was performed under general anesthesia. To prevent surgical site infection, all patients received 1 g cefazolin 1 h before the surgical incision was made. Under direct view, a 5-cm vertical paramedian incision was made 2 cm below the umbilicus and 2 cm to the right or left of the midline. The underlying subcutaneous tissues were opened until the anterior rectus sheath was exposed. The muscle fibers were bluntly separated to reach the posterior rectus sheath. The fascia of the posterior rectus sheath and the peritoneum were lifted and incised to enter the abdominal cavity. A straight PD catheter with double cuffs (Long Shoot Catheter 650 with reinforcement JL-1(A)S®; Hayashidera Medinol, Kanazawa, Japan) was inserted with an internal stiffening stylet. The internal cuff was fixed to the posterior sheath and the peritoneum with 2–0 coated braided polyester sutures (Ti-Cron™; Covidien Japan, Tokyo, Japan) after testing flow function by injecting 100 mL normal saline solution. To prevent fluid leakage, a strong purse-string suture was made at the level of the posterior muscle and the peritoneum. The PD catheter was then directed through a subcutaneous tunnel to the previously marked skin exit site using a tunneler. All skin exit sites were directed downward or laterally (not upward). Confirmation of successful fill and drain of 1000 mL dialysate was followed by closure of the vertical paramedian incision. All patients were administered 2 g cefazolin daily for 3 days after PD catheter insertion.

### Postoperative procedures

Patients assigned to the NPWT group received treatment delivered through the vacuum-assisted closure device (VAC® Therapy System; KCI, Tokyo, Japan) immediately after the operation. The exit site was covered with special foam dressings (VAC® GranuFoam™ Dressing; KCI) and sealed with a clear drape (VAC® Drape; KCI) that helped maintain negative pressure over the site. One end of the tube connected to the dressing through the pressure-sensing pad (SENSAT.RAC™ Pad; KCI), and the other end connected to the VAC® Therapy Unit and Canister system (KCI). Once connected to the device, a continuous negative pressure of 125 mmHg was applied in accordance with the instruction manual. NPWT was administered at the exit sites, and the dressing was left in situ for 7 days until the patients underwent first assessment of the site.

Patients assigned to standard care (the non-NPWT group) were treated using waterproof see-through film dressings. When filled with exudate, the dressings were changed appropriately.

### Daily exit-site care and follow-up

Over a three-week hospitalization period, the patients underwent PD training conducted by nursing staff with appropriate qualifications and experience in accordance with the ISPD guidelines^5^. The patients were taught to begin exit-site care and fluid exchange with hand washing and mask wearing. After examining the sites by themselves, the patients were trained to clean each site with a cotton swab soaked in 0.05% chlorhexidine and to cover with sterile gauze. The PD catheter was properly fixed to the abdominal wall by gentle taping onto the skin to avoid traction, which could cause injury and bleeding.

After discharge, the patients underwent monthly assessment of the exit site conducted by the attending doctor and nursing staff. The exit site was also photographed for further evaluation. The patients were followed up for 180 days from the day of PD catheter insertion.

### Study outcomes

The primary outcome of the present study was exit-site score on the seventh postoperative day. The secondary outcomes were exit-site score over 180 days, time to first CRI, time to first PD-related peritonitis, and catheter loss due to PD-related infections.

The exit-site score was calculated according to the ISPD guidelines^5^. The exit-site scoring system, which was first proposed by Schaefer et al*.* (Table 2)^26^, is recommended for evaluating and monitoring exit sites^32^. ESI was defined as the presence of purulent discharge with or without erythema of the skin at the catheter–epidermal interface^5^. Tunnel infection was defined as the presence of clinical inflammation or ultrasonographic evidence of collection along the catheter tunnel^5^. Peritonitis was diagnosed based on the presence of fever or abdominal pain and cloudy effluent (after a dwell time of at least 2 h) with a white blood cell count ≥ 100/μL, comprising ≥ 50% polymorphonuclear cells^1^.

**Table 2:** Exit-site scoring system.

	0 points	1 point	2 points
Swelling	No	< 0.5 cm	≥ 0.5 cm
Crust	No	< 0.5 cm	≥ 0.5 cm
Redness	No	< 0.5 cm	≥ 0.5 cm
Pain	No	Slight	Severe
Drainage	No	Serous	Purulent

### Statistical analysis

The sample size estimate was calculated for the primary outcome of exit-site score on the seventh postoperative day, by nonparametric analysis using the wmwpow package. In the non-NPWT group, the rates of patients with exit-site scores of 0, 1, and 2 were 0%, 50%, and 50%, respectively. In contrast, in the NPWT group, the rates of patients with exit-site scores of 0, 1, and 2 were 30%, 60%, and 10%, respectively. In total, 15 patients were recruited for each group after accounting one patient for loss to follow-up. With an alpha error of 0.05 and a beta error of 0.2 (80% power), the required sample size was 14 patients in each group.

The NPWT group was compared to the non-NPWT group. The Mann–Whitney *U* and Fisher’s exact tests were used to compare baseline quantitative and categorical variables, respectively, between the two groups. Data were presented as medians (minimum value–maximum value) unless otherwise stated. The primary endpoint was analyzed using the Mann–Whitney *U* test, analysis of variance was used for all observational periods of the exit-site score, and the Kaplan–Meier analysis was used for event-free analysis.

Statistical analyses were performed using SAS software (version 9.4, SAS Institute, Cary, NC) and R statistical software package (R version 4.0.3). R package is an open-source environment for statistical data analysis, and it is readily available (nparLD package, mice package). A p value of < 0.05 was considered to indicate statistical significance.

## Data Availability

The datasets analyzed in this study are available from the corresponding author on reasonable request.
